# *In silico* design of novel dihydropteridone derivatives with oxadiazoles as potent inhibitors of MCF-7 breast cancer cells

**DOI:** 10.3389/fchem.2025.1590593

**Published:** 2025-07-28

**Authors:** Mourad Aloui, Mohamed El fadili, Mohammed Er-rajy, Somdutt Mujwar, Hatem A. Abuelizz, Sara Er-rahmani, Elhalaoui Menana

**Affiliations:** ^1^LIMAS Laboratory, Faculty of Sciences Dhar El Mahraz, Sidi Mohamed Ben Abdellah University, Fez, Morocco; ^2^Chitkara College of Pharmacy, Chitkara University, Rajpura, Punjab, India; ^3^Department of Pharmaceutical Chemistry, College of Pharmacy, King Saud University, Riyadh, Saudi Arabia; ^4^Dipartimento di Chimica, Universita’ di Torino, Torino, Italy

**Keywords:** QSAR, molecular docking, oxadiazole, molecular dynamics, ADMET propriety, MCF-7, breast cancer cells

## Abstract

**Introduction:**

Pharmaceutical treatment protocols or combination therapies based on chemical compounds make it possible to target cancer cells, which can be complicated by several factors, including their resistance to bioactive compounds and the potential for drugs to damage certain healthy cells.

**Methods:**

This project was designed to assess the structural relationship between new dihydropteridone-derived compounds bearing an oxadiazole moiety and their corresponding cytotoxicity against breast cancer, using computational chemistry tools. The aim of this research is to better understand how compound properties influence their activity and to understand the underlying mechanisms, which could then be integrated into the anticancer drug design process with a view to recommending new optimized compounds likely to have the desired activity.

**Results and discussions:**

The results show that the predicted molecules possess enhanced selective cytotoxic inhibitory activity against breast cancer cells (MCF-7). Guided by these analyses, we designed five novel dihydropteridone derivatives incorporating an oxadiazole moiety. These compounds exhibited favorable interactions with key breast cancer-related proteins, demonstrated enhanced dynamic stability within their binding sites, and adhered to established drug-likeness principles. Importantly, these compounds displayed promising oral absorption (88%) in preliminary assessments and exhibited no significant toxicity. These findings suggest that these novel dihydropteridone-oxadiazole derivatives warrant further investigation as potential multifunctional agents for the treatment of breast cancer cells (MCF-7).

## Introduction

Breast cancer is the most common cancer affecting women worldwide, representing approximately 24.5% of all new cancer cases diagnosed in women in 2022. According to the World Health Organization (WHO) and the International Agency for Research on Cancer (IARC), in 2022, an estimated 2.3 million women were diagnosed with breast cancer, and tragically, 670,000 succumbed to the disease globally (Breast cancer, 2025; Cancer, 2024; Cancer Today, 2025; Information and Resources about Cancer: Breast, Colon, Lung, Prostate, Skin, 2025). Despite advancements in treatments like surgery, chemotherapy, and hormone therapy, many patients experience systemic toxicity due to the non-selective nature of these approaches (Zargan et al., 2022; CDC, 2024). Additionally, drug resistance remains a significant obstacle in breast cancer treatment, limiting the effectiveness of current therapies (Chemoresistance mechanisms of breast cancer and their countermeasures - ScienceDirect, 2024).

Given the limitations of current breast cancer treatments, developing innovative drugs that can overcome these challenges is crucial. One promising avenue is the exploration of dihydropteridone derivatives containing oxadiazoles (Li et al., 2023; Hou et al., 2025), which have demonstrated potential as alternatives to existing therapies due to their antitumor properties and selectivity (Aloui et al., 2024b; Analyzing the Cytotoxic and Genetic Impact of Datura stramonium Extract on MCF7 and HT29 Cancer Cells: A Metabolite and Gene Expression Study, 2025). This study employs quantitative structure-activity relationship (QSAR) methods to quantitatively analyze the relationship between the structure of these compounds and their anticancer activity, facilitating the design of more potent and effective inhibitors of MCF-7 cells (Bailly, 2012; Kojja et al., 2025). The present study is based on a multifaceted approach, encompassing QSAR modeling, molecular docking and molecular dynamics simulation, to identify and optimize novel MCF-7 inhibitors (Sun et al., 2008; Hassan et al., 2025). Using molecular descriptors such as lipophilicity and geometry, QSAR models predict the activity of potential inhibitors on the basis of their structural features (Danishuddin and Khan, 2016). Molecular docking analysis is employed to determine the optimal binding mode between the ligand and the target protein, shedding light on key interactions (Zhang et al., 2022). To delve deeper into these interactions and assess their stability, molecular dynamics (MD) simulations are conducted over a 100 ns timescale, examining the protein-ligand complex using newly designated, highly active molecules (Binary quantitative activity-activity relationship QAAR studies to explore selective HDAC8 inhibitors: In light of mathematical models, DFT-based calculation and molecular dynamic simulation studies - ScienceDirect, 2024). In addition to this, *in silico* studies are being carried out on ADMET to assess the new compound’s potential as an anti-cancer drug and predict its pharmacokinetic and toxicological profiles. This will enable the development of new, better-targeted drugs more suited to the fight against breast cancer. Structural modifications of new dihydropteridone derivatives possessing oxadiazoles can significantly increase their bioactivity (Li et al., 2023). Furthermore, the research group reported that compound M5 is highly selective towards the MCF-7 cell line and displays low toxicity towards healthy breast cells.

The aim of this introduction is to highlight the central role of QSAR methods in the search for novel anticancer agents for the treatment of breast cancer, in order to find effective and innovative pharmaceutical compounds. Molecular docking studies have been carried out with these compounds to better understand the key structural requirements and interactions between the ligand and the 2RKU.pdb protein (Aloui et al., 2024a). The results of these simulations were promising and aligned well with experimental data. Finally, 100 ns MD simulations are carried out to estimate ligand-receptor stability under normal physiological conditions. All molecules designed are also examined using conventional computational pharmacokinetic parameters (ADMET) and pharmacokinetics to assess their pharmacological potential. This approach speeds up the pace of new drug discovery, provides a better understanding of structure-activity relationships, and paves the way for therapies that are both more effective and better tolerated. This project is divided into two chapters: the first describes the materials and methods used in this research, followed by another chapter devoted to a detailed discussion of the results obtained from computer simulations, and the final section summarizes the main findings and overall implications of this research.

## Materials and methods

### Experimental dataset

Experimental data on 33 novel dihydropteridone compounds with an oxadiazole moiety that are effective MCF-7 inhibitors are shown in the following table (Table 1).

**TABLE 1:** Dihydropteridone derivatives’ structures and pIC_50_ values as powerful PLK1 inhibitors.

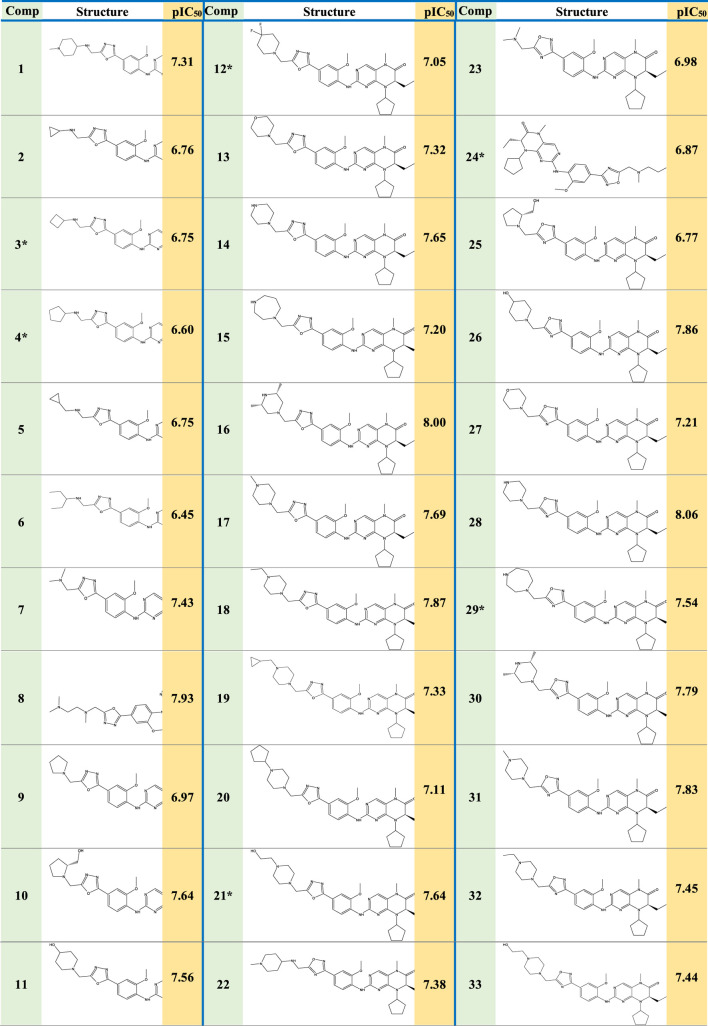

*Refer to test set molecules. pIC_50_ = 6-log_10_ (IC_50_).

### Examined compounds

Based on experimental data on the inhibitory activities of 33 previously synthesized dihydropteridone-derived molecules containing an oxadiazole moiety against MCF-7 cells, we conducted molecular modeling studies (Li et al., 2023). To standardize and simplify the analysis, the observed activity values (IC_50_) were transformed into pIC_50_ values, using a logarithmic scale of log (IC_50_), the values of which are shown in Table 1.

### Molecular descriptor calculation for investigated compounds

A set of 17 descriptors, including geometric properties, lipophilicity, physicochemical attributes and steric characteristics, were employed to develop a robust QSAR model.

Molecular descriptors were determined using the MM2 method integrated into the ChemBioOffice and ACD/ChemSketch (Österberg and Norinder, 2001; Milne, 2010). Our approach to molecular geometry was optimized by DFT calculations based on the B3LYP/6-311G (d,p) basis set (Parr and Yang, 1995; Zhang et al., 2009). All electronic descriptors were determined using Gaussian 09 quantum chemistry software (Frisch et al., 2004). The calculated descriptor values are summarized in Table 2.

**TABLE 2:** Descriptor values for the 33 compounds and observed activity.

Comp	S	B	S-B	Tor	LogP	NHBA	NHBD	NRB	MP	TD	MV	IR	ST	E_HOMO_	E_LUMO_	Repul	η	pIC_50_
1	3.17	26.86	0.57	5.34	3.12	11	2	9	1221.15	21	434.9	1.645	72	−0.1961	−0.0486	4588.56	0.07378	7.31
2	2.57	26.23	0.32	5.49	3.47	10	2	9	1161.91	18	380.6	1.657	75.2	−0.1966	−0.0493	3983.94	0.07366	6.76
3*	3.32	41.93	−0.61	14.46	3.89	10	2	9	1169.66	19	396.6	1.649	73.3	−0.1964	−0.0490	4127.68	0.07371	6.75
4*	2.86	27.88	0.26	9.60	4.30	10	2	9	1177.41	19	412.7	1.643	71.7	−0.1961	−0.0485	4278.85	0.07379	6.60
5	2.77	27.24	0.16	7.36	3.90	10	2	10	1173.18	19	416.7	1.62	60.2	−0.1883	−0.0408	4108	0.07378	6.75
6	2.99	27.40	0.53	5.95	4.80	10	2	11	1151.51	19	453.8	1.589	54	−0.1960	−0.0483	4317.95	0.07381	6.45
7	2.86	26.19	0.50	2.94	3.55	10	1	8	1112.51	17	402.4	1.609	57.9	−0.1964	−0.0493	3916.85	0.07355	7.43
8	3.62	25.48	0.73	8.41	3.55	11	1	11	1178.79	20	459.1	1.599	56.1	−0.1965	−0.0487	4466.09	0.0739	7.93
9	3.06	28.26	0.32	10.84	3.87	10	1	8	1153.71	18	414.4	1.624	61.5	−0.1955	−0.0479	4206.83	0.0738	6.97
10	3.25	29.82	0.42	10.76	3.33	11	2	9	1221.56	19	433	1.622	62.2	−0.1956	−0.0481	4533.19	0.07375	7.65
11	3.16	26.83	0.58	5.57	2.98	11	2	8	1218.04	20	429.2	1.629	64.4	−0.1962	−0.0492	4538.53	0.07348	7.56
12*	3.20	26.39	0.57	4.90	4.46	12	1	8	1182.3	20	421	1.629	69.5	−0.1987	−0.0526	4727.18	0.07305	7.05
13	3.22	28.09	0.70	6.24	3.15	11	1	8	1176.76	19	423.2	1.616	60.7	−0.1969	−0.0498	4381.84	0.07354	7.32
14	3.06	26.23	0.53	1.81	2.93	11	2	8	1255.22	19	428.2	1.616	59.4	−0.1962	−0.0489	4373.21	0.07362	7.65
15	3.16	31.59	0.79	10.17	3.03	11	2	8	1262.97	19	445.9	1.608	57.7	−0.1944	−0.0464	4577.86	0.07402	7.20
16	3.26	28.25	0.63	5.46	3.57	11	2	8	1269.28	19	468.2	1.595	53.3	−0.1966	−0.0496	4745.28	0.07349	8.00
17	3.31	26.82	0.64	1.80	3.31	11	1	8	1197.45	20	443.2	1.615	58.8	−0.1965	−0.0494	4532.35	0.07355	7.69
18	3.47	27.34	0.69	2.16	3.64	11	1	9	1208.72	21	460.8	1.607	57.2	−0.1964	−0.0493	4693.53	0.07354	7.87
19	3.68	29.28	0.48	5.31	4.03	11	1	10	1249.2	22	470.1	1.626	61.4	−0.1959	−0.0488	5007.98	0.07354	7.33
20	3.81	29.57	0.62	8.63	4.44	11	1	9	1253.43	22	485.6	1.622	60.8	−0.1963	−0.0490	5170.85	0.07361	7.11
21*	3.48	27.74	0.71	2.16	2.79	12	2	10	1269.54	22	458.3	1.619	61.6	−0.1969	−0.0499	4852.38	0.0735	7.64
22	3.16	29.95	0.51	7.45	4.23	11	2	9	1221.15	21	434.9	1.645	72	−0.1940	−0.0450	4625.67	0.07451	7.38
23	2.85	29.07	0.44	5.35	4.66	10	1	8	1112.51	17	402.4	1.609	57.9	−0.1936	−0.0454	3894.38	0.07408	6.98
24*	3.53	31.73	0.78	6.57	4.66	11	1	11	1178.79	20	459.1	1.599	56.1	−0.1937	−0.0458	4544.13	0.07395	6.87
25	3.24	32.64	0.36	13.26	4.44	11	2	9	1221.56	19	433	1.622	62.2	−0.1937	−0.0451	4502.94	0.07428	6.77
26	3.14	29.69	0.52	8.07	4.09	11	2	8	1218.04	20	429.2	1.629	64.4	−0.1933	−0.0449	4512.91	0.07418	7.86
27*	3.21	30.93	0.64	8.72	4.26	11	1	8	1176.76	19	423.2	1.616	60.7	−0.1944	−0.0466	4336.63	0.07393	7.21
28	3.04	29.10	0.47	4.27	4.04	11	2	8	1255.22	19	428.2	1.616	59.4	−0.1935	−0.0451	4340.67	0.0742	8.06
29*	3.16	34.41	0.73	12.58	4.14	11	2	8	1262.97	19	445.9	1.608	57.7	−0.1915	−0.0417	4539.71	0.07494	7.54
30	3.26	31.32	0.59	7.70	4.67	11	2	8	1269.28	19	468.2	1.595	53.3	−0.1937	−0.0450	4756.57	0.07437	7.79
31	3.30	29.72	0.58	4.26	4.42	11	1	8	1197.45	20	443.2	1.615	58.8	−0.1934	−0.0446	4524.05	0.07441	7.83
32	3.46	30.25	0.64	4.60	4.75	11	1	9	1208.72	21	460.8	1.607	57.2	−0.1934	−0.0445	4691.05	0.07444	7.45
33	3.49	30.73	0.66	4.50	3.90	12	2	10	1269.54	22	458.3	1.619	61.6	−0.1937	−0.0451	4849.24	0.07431	7.44

S, Stretch energy (kcal/mol); T, Torsion energy (kcal/mol); B, Bend energy (kcal/mol); NHBD, number of hydrogen bond Donors; S-B, Stretch-Bend energy (kcal/mol); NHBA, number of hydrogen bond acceptors; NRB, number of Rotate Band; MP, Melting Point; TD, ; MV, Molar Volume (cm^3^); E_HOMO_, Energy of the Highest occupied molecular orbital (Hartree); IR, Index of Refraction; ST, Surface Tension (dyne/cm); repul, repulsion; E_LUMO_, Energy of the lowest unoccupied molecular orbital (Hartree); η, hardness (Hartree).

### QSAR modeling

A set of 33 compounds was chosen from earlier studies that shown remarkable activity as new inhibitors of MCF-7 in the quest of developing a QSAR model. To construct the QSAR model. The dataset was randomly divided into two subsets: a training set of 26 molecules for model development and a test set of seven molecules for validation. Multiple linear regression (MLR) and artificial neural networks (ANN) were employed as modeling techniques (Gupta et al., 2016).

### Multiple linear regression

MLR is a widely used technique in QSAR studies, due to its simplicity and reliability when selecting molecular descriptors (Roy and Mitra, 2011). MLR is often combined with other methods, notably multinomial nonlinear regression (MNLR) and ANN. to identify relevant descriptors for building QSAR models. MLR models assume a linear relationship between the dependent variable and a set of independent variables as described by the following equation (Equation 1).

$$Y=a_{0}+\sum_{i=1}^{n}a_{i}X_{i}$$ (1)

Y is the biological activity. Xi the molecular descriptors. a_0_ is the intercept and ai are the coefficients associated with each descriptor.

### Multiple nonlinear regression

The MNLR model is a non-linear method used to establish the relationship between molecular descriptors (Xi) and the corresponding biological activity (Y). It identifies the optimal mathematical representation of this non-linear variation (Er-rajy et al., 2022b). In this scenario. a second-order polynomial model is employed for constructing the QSAR model using the MNLR method based on descriptors from MLR models. The MNLR linking these molecular descriptors with biological activity could be modeled by the following equation (Equation 2):

$$Y=a_{0}+\sum_{i=1}^{n}a_{i}{\times X}_{i}+b_{i}\times X_{i}^{2}$$ (2)

In this equation, Y represents the predicted biological activity. Xi are the molecular descriptors. n is the number of descriptors and a_0_, ai, and bi are the model coefficients.

### Artificial neural networks

**FIGURE 1:**
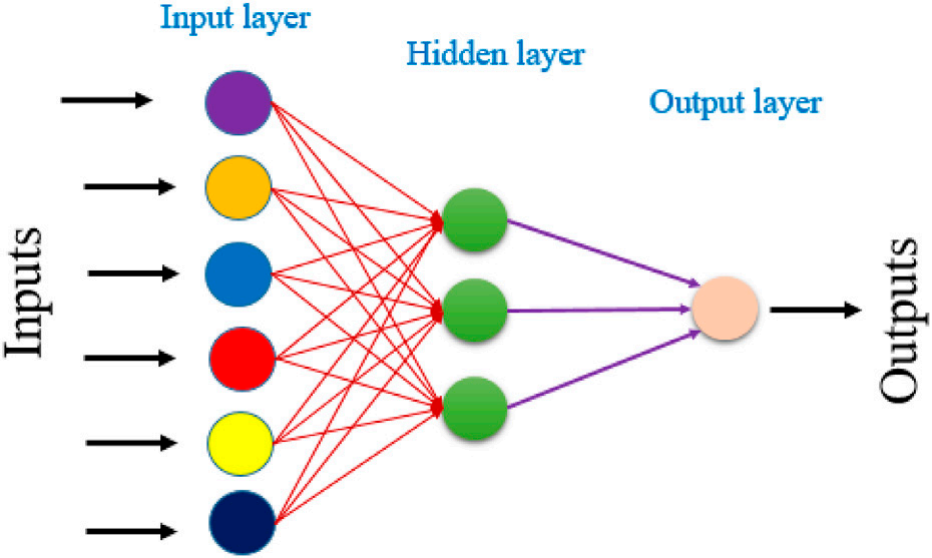
The architecture of the ANN model used in this study.

To enhance the ability to characterize compounds and predict biological activity. We developed an ANN-based QSAR model (Aloui et al., 2025). The ANN model was developed using the molecular descriptors identified through MLR analysis. The network architecture consisted of three layers: input hidden and output (Figure 1).

The input neural network corresponded to that of the descriptors. While the output layer predicted biological activity. We optimized the ANN’s performance by precisely determining the number of neurons in the hidden layer and the learning rate (ρ). To ensure model reliability and statistical validity. ρ was maintained within the recommended range of 1–3 (Kůrková, 1992).

### Cross-validation (CV)

To assess the accuracy of the QSAR models developed using MLR, MNLR and ANN. We use Leave-One-Out cross-validation (LOO-CV) (Golbraikh and Tropsha, 2002). In this method, each molecule is removed from the dataset in turn, so that the model is retrained and then used to predict the activity of that molecule. This operation is repeated for all molecules (Roy and Mitra, 2011). The $R_{\text{cv}}^{2}$ coefficient, as calculated by Equation 3, is used to evaluate the model’s performance. An $R_{\text{cv}}^{2}$ value above 0.5 generally indicates a robust model (Golbraikh and Tropsha, 2002).

$$R_{\text{cv}}^{2}=1‐\frac{\sum \left(Y_{\text{ob}}\left(\text{trai}\right)‐Y_{\text{ca}}\left(\text{trai}\right)\right)2}{\sum \left(Y_{\text{Ob}}\left(\text{trai}\right)‐\bar{Y_{\text{trai}}}\left(\text{trai}\right)\right)2}$$ (3)

$Y_{\text{ob}}\left(train\right)$ represents the experimentally observed biological activity in the training set. $Y_{\text{ca}}\left(trai\right)$ denotes the predicted biological activity obtained using the LOO-CV technique on the training set. $\bar{Y_{trai}}\left(trai\right)$ represents the average of both the observed and predicted biological activities in the training set.

### Applicability domain

The applicability domain (AD) defines the region in chemical space where a QSAR model can reliably predict the activity of compounds. Compounds falling outside this domain may have less reliable predictions (Gramatica, 2007). To assess AD leverage values (hi) are calculated for each compound using Equation 4:

$$h_{i}=x_{i}{\left(X^{T}X\right)}^{-1}x_{i}^{T}\;i=1.2.\ldots ...n$$ (4)

where xi is the vector of descriptors for a query compound. X is the descriptor matrix of the training set, and n is the number of compounds. If h_i_ exceeds the critical value (h* = 3 (p + 1)/n, where p is the number of variables), the compound is considered outside the AD. Conversely, a leverage value below h* suggests a high likelihood of accurate predictions.

### Molecular docking

In the present work, a molecular docking process was carried out with the assistance of Autodock 4.2 and Discovery Studio 2021 programs, in which the most active compound (C28) and the candidate ligand (M5) were chosen to be docked towards the structure of PLK1 in complex with BI2536 (PDB ID of 2RKU) as a targeted receptor responsible to the cytotoxic inhibitory activity against breast cancer cells (MCF-7). In the first stage, the targeted protein was prepared by adding the charges of Gasteiger, removing Water (H_2_O) molecules and all suspended ligands (El fadili et al., 2023a; Ed-Dahmani et al., 2024; Nouioura et al., 2024a). Then, C28 and M5 were docked the prepared protein using Autodock4.2 software (El fadili et al., 2023c; Nouioura et al., 2024b). In the second stage, Discovery Studio 2021 software was employed to visualize the produced contacts in two and three dimensions (El fadili et al., 2023b; El fadili et al., 2024).

### Molecular dynamics

A molecular dynamics (MD) technique was equally performed to examine the thermodynamic stability of the produced intermolecular contacts throughout 100 nanoseconds of MD simulation time using Desmond, a package of LLC Schrödinger software, in which the output files of molecular docking were chosen as input files of molecular dynamics, working on standardized physiological conditions with a Pressure of 1 atm, Temperature of 300 K, OPLS force fields, and Counter ions in 0.15 M salt (Na+, Cl) (El fadili et al., 2022; Er-rahmani et al., 2024).

### *In silico* pharmacokinetic-pharmacodynamic modeling

Due to advances in computer technology, drug design has been accelerated by reducing the number of in-depth experiments. Early identification of ADMET properties and drug similarity is now crucial in the drug discovery process. *In silico* methods make it possible to accurately assess the main ADMET parameters, including absorption, distribution, metabolism, excretion and toxicity (Vickers, 2017).

Lipinski’s Rule of Five is a valuable tool for predicting drug-likeness. Compounds violating two or more of these rules often exhibit challenges in ADMET properties (Hansch et al., 2004). Notably, nearly 10% of drugs reaching clinical trials do not adhere to these rules. Beyond Lipinski’s rule, factors such as topological polar surface and number of rotational bonds also influence drug similarity (Jin et al., 2020). Predicting these factors helps us understand the flexibility of molecular interactions with receptors.

## Results and discussion

### 2D-QSAR study

Numerous tests were carried out to create a reliable model once the molecular descriptors of 33 derivatives had been calculated (Table 2). The six descriptors Stretch, Torsion, log P, Number of rotating bonds, Molar volume and hardness were used to construct the most appropriate model. Based on the results, the following molecules (3, 4, 12, 21, 24 and 29) were chosen for the test set. In addition the following molecules (1, 2, 5, 6, 7, 8, 9, 10, 11, 13, 14, 15, 16, 17, 18, 19, 20, 22, 23, 25, 26, 27, 28, 30, and 31) were chosen for the training set. Equation 5 shows the QSAR model created by the MLR approach.

$${\text{pIC}}_{50}=-10.59993+0.81144\times S-0.09127\times \text{Tor}+0.00643\times \text{MP}-0.24116\times \text{NRB}-0.00086\times \text{repul}+191.40275\times \eta$$ (5)

$$N=26;\;R2=0.91;\;{\text{MSE}=0.020\;;\;R=0.95\;;\;R}_{\text{Ajus}}^{2}=0.88;\mathrm{Pr}<\;0.\;0001;\;F=30.363;\;R_{cv}^{2}=0.83$$

is the number of compounds in the training set. While MSE corresponds to mean square error.

As shown in Equation 5, MCF-7 inhibitory biological activity (pIC_50_) values correlate linearly with the five selected descriptors. The following parameters are used to evaluate the QSAR model developed using the MLR technique: *R* ^2^, F, MSE, P-value and ${\;R}_{\text{cv}}^{2}$ .

These statistical measures, including a high coefficient of determination (*R* ^2^ = 0.91), a low root-mean-square error (MSE = 0.02) and a high F-statistic (F = 30.36), reveal a good statistical performance of the QSAR model presented in Equation 5. This suggests that the model is capable of accurately describing the relationship between molecular descriptors and biological activity. Additionally, the calculated P-value (Pr < 0.0001) confirms the statistical significance of the model’s equation at a confidence level above 95%.

The observed biological activity against MCF-7 breast cancer cells can be mechanistically explained through the combined influence of several molecular descriptors. Stretch and Torsion describe the internal flexibility of the molecules and their ability to adopt bioactive conformations within the survivin binding pocket; lower energy values for these parameters enhance molecular adaptability and binding affinity. The log P value reflects lipophilicity, which is crucial for cell membrane permeability and intracellular accumulation—a balanced log P ensures sufficient solubility and effective penetration into MCF-7 cells. The number of rotating bonds represents molecular flexibility; moderate flexibility facilitates optimal conformational adjustments for target binding without compromising structural stability. Molar volume affects the steric fit within the survivin binding site—an appropriate volume allows efficient cavity occupation and maximization of molecular interactions. Finally, hardness, related to the molecule’s electronic properties, indicates its potential for stabilizing interactions with the target protein; softer molecules (lower hardness) generally display enhanced electronic reactivity, favoring biological activity. Altogether, these descriptors provide a rational explanation for the enhanced antiproliferative activity observed in MCF-7 cells.

**FIGURE 2:**
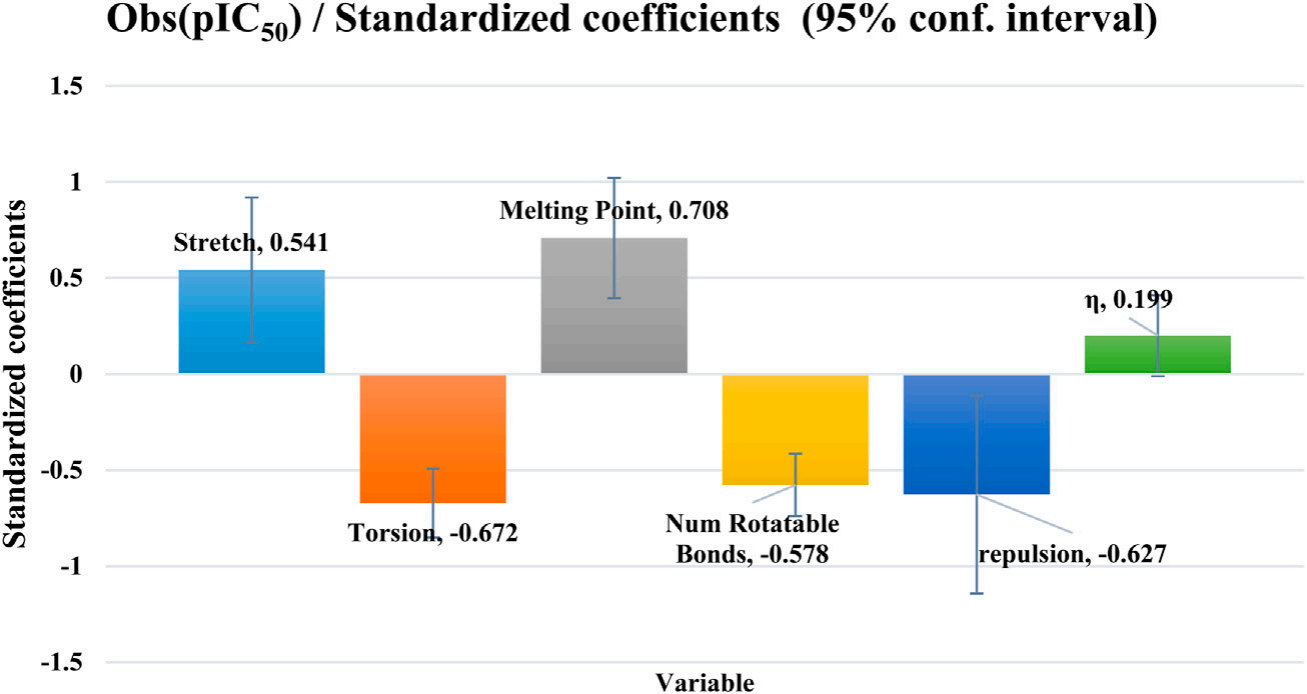
The contribution coefficients of the five molecular descriptors of the MLR model.

Stretch, melting point and hardness have a favorable effect on biological activity, while torsion, repulsion and the number of rotational bonds have an unfavorable effect, as shown in Figure 2. The cross-validation correlation coefficient ( $R_{cv}^{2}$ = 0.83) is well above the 0.5 threshold, confirming the robustness of the QSAR model derived from MLR.

**FIGURE 3:**
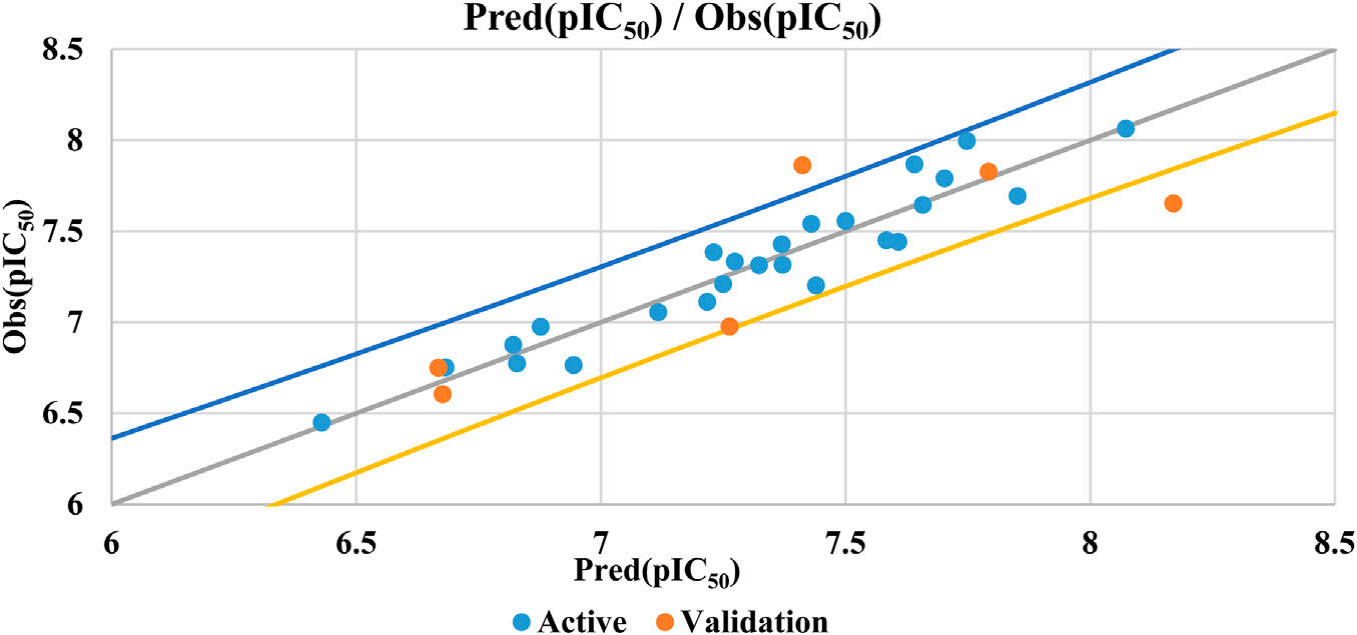
MLR model correlations between predicted and observed activity values.

The presence of an $R_{cv}^{2}$ value below *R* ^2^ indicates the model’s sensitivity when an element is omitted from the training set. Figure 3 illustrates the correlation between observed and predicted activities demonstrating the QSAR model’s ability to accurately predict the biological activity of both training and test set molecules. Figure 3 highlights the perfect correlation between predicted and observed pIC_50_ values, confirmed by a low root-mean-square error (RMSE). The formula in Equation 5 groups together the five descriptors showing a significant linear correlation with biological activity. To enhance alignment between the QSAR model’s predicted activities and the six molecular descriptors (S, Tor, MP, NRB, repul and η) we can consider incorporating additional descriptors or refining the existing ones, a new QSAR model is formulated employing two non-linear methodologies: MNLR and ANN techniques.

### Multiple nonlinear regression

The QSAR model, developed using the MNLR technique, is presented in Equation 6:

$${pIC}_{50\;}=1166+7.30934\times S-0.04749\times \text{Tor}+0.02265\times \text{MP}+0.14423\times \text{NRB}-0.00686\times \text{repul}-31788\times \eta -0.97489\times S^{2}-0.00283\times {\text{Tor}}^{2}-6.13750\times 10^{-6}\times {\text{MP}}^{2}-0.01803\times {\text{NRB}}^{2}+6.14639E-7\times {\text{repul}}^{2}+215698\times \eta^{2}$$ (6)

$N=25\;;\;R=0.96\;;\;R^{2}=0.93\;;\;MSE=0.025$

The QSAR model’s performance is validated by its strong coefficient of determination (*R* ^2^) of 0.93, low mean squared error (MSE) of 0.025, and high correlation coefficient (R) of 0.96. These metrics indicate the model’s statistical significance and its ability to accurately predict biological activity. The biological activities predicted by the QSAR model, based on both linear and non-linear regression, are presented in Table 3 for the training and test sets.

**TABLE 3:** Comparison of observed and predicted biological activities using the QSAR model.

Comp	S	Tor	MP	NRB	repul	η	pIC_50_Obs	MLR		MNLR	
								pIC_50_Pred	*Resid*	pIC_50_Pred	*Resid*
1	3.1684	5.3358	1221.15	9	4588.56	0.073775	7.31	7.324	−0.011	7.345	−0.032
2	2.5717	5.4927	1161.91	9	3983.94	0.07366	6.76	6.944	−0.180	6.793	−0.028
3*	3.3203	14.4563	1169.66	9	4127.68	0.073705	6.75	6.668	0.082	6.829	−0.080
4*	2.8648	9.6019	1177.41	9	4278.85	0.07379	6.60	6.677	−0.073	6.618	−0.014
5	2.7726	7.3625	1173.18	10	4108.00	0.073775	6.75	6.683	0.069	6.721	0.031
6	2.9858	5.9475	1151.51	11	4317.95	0.07381	6.45	6.430	0.019	6.436	0.012
7	2.8589	2.9396	1112.51	8	3916.85	0.073545	7.43	7.370	0.058	7.425	0.004
8	3.0643	10.8436	1153.71	8	4206.83	0.073795	6.97	6.877	0.097	6.875	0.100
9	3.1563	5.5693	1218.04	8	4538.53	0.07348	7.56	7.500	0.056	7.521	0.035
10	3.1966	4.904	1182.3	8	4727.18	0.073045	7.05	7.118	−0.063	7.195	−0.140
11	3.2185	6.2351	1176.76	8	4381.84	0.07354	7.32	7.371	−0.056	7.417	−0.102
12*	3.0551	1.8107	1255.22	8	4373.21	0.07362	7.65	8.170	−0.518	8.148	−0.497
13	3.1579	10.1711	1262.97	8	4577.86	0.07402	7.20	7.440	−0.238	7.399	−0.197
14	3.2615	5.4632	1269.28	8	4745.28	0.07349	8.00	7.748	0.248	7.780	0.216
15	3.3069	1.8001	1197.45	8	4532.35	0.073545	7.69	7.852	−0.159	7.772	−0.080
16	3.4701	2.1641	1208.72	9	4693.53	0.073535	7.87	7.641	0.225	7.602	0.265
17	3.6827	5.3083	1249.2	10	5007.98	0.073535	7.33	7.274	0.058	7.284	0.048
18	3.8117	8.632	1253.43	9	5170.85	0.07361	7.11	7.218	−0.106	7.124	−0.012
19	3.4844	2.1634	1269.54	10	4852.38	0.073495	7.64	7.658	−0.014	7.709	−0.065
20	3.1592	7.4478	1221.15	9	4625.67	0.074505	7.38	7.231	0.152	7.256	0.127
21*	2.85	5.3534	1112.51	8	3894.38	0.074075	6.98	7.263	−0.287	7.312	−0.337
22	3.5298	6.5685	1178.79	11	4544.13	0.07395	6.87	6.821	0.053	6.827	0.048
23	3.238	13.2614	1221.56	9	4502.94	0.074275	6.77	6.829	−0.055	6.811	−0.037
24*	3.138	8.0742	1218.04	8	4512.91	0.074175	7.86	7.412	0.450	7.359	0.502
25	3.208	8.7184	1176.76	8	4336.63	0.07393	7.21	7.250	−0.041	7.260	−0.050
26	3.0406	4.2684	1255.22	8	4340.67	0.0742	8.06	8.073	−0.010	8.074	−0.011
27	3.1589	12.5753	1262.97	8	4539.71	0.07494	7.54	7.430	0.110	7.494	0.047
28	3.2619	7.7031	1269.28	8	4756.57	0.07437	7.79	7.703	0.088	7.672	0.119
29*	3.298	4.2607	1197.45	8	4524.05	0.07441	7.83	7.793	0.034	7.725	0.102
30	3.4583	4.5979	1208.72	9	4691.05	0.074435	7.45	7.584	−0.134	7.552	−0.102
31	3.491	4.4959	1269.54	10	4849.24	0.074305	7.44	7.608	−0.167	7.635	−0.194

The high coefficient of determination ( $R_{\text{cv}}^{2}$ = 0.83) obtained through LOO-CV confirms the robustness of the non-linear model. This indicates that all 25 training set components contributed significantly to the model’s effectiveness and reliability

**FIGURE 4:**
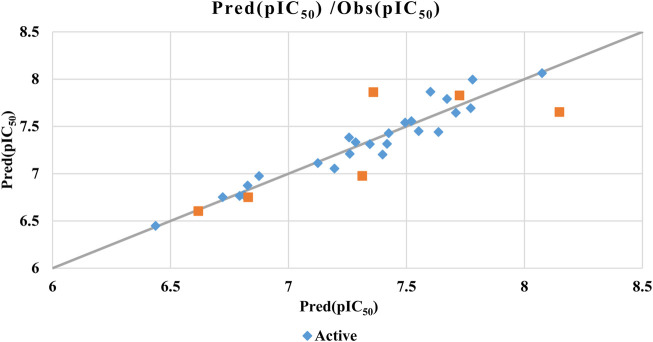
Correlations between observed and predicted activity using the MNLR model.

Figure 4 demonstrates the strong correlation between experimental and predicted pIC_50_ values, indicating the high accuracy of the QSAR model.

### Artificial neural networks

The ANN technique is used to generate a QSAR model a 6-three to one architecture is utilized with a parameter ρ set to 1. The proportion of the number of neurons in the hidden layer (3) to the number of descriptors in the input layer (6) is reflected by the value of ρ, which typically ranges between 1 and 3. This architecture aids in predicting the pIC_50_ values represented by the single neuron in the output layer. The QSAR model, developed using the ANN technique, exhibited exceptional performance, as evidenced by a coefficient of determination (*R* ^2^) of 1 and a mean squared error (MSE) of 0.0005. These results strongly suggest the model’s statistical significance and its ability to accurately predict antiproliferative inhibitory activity against MCF-7 cancer cells. Consequently, predicting pIC_50_ values using the six selected descriptors, namely, S, Tor, MP, NRB, repul and η is highly relevant. These descriptors were chosen specifically because they were deemed suitable for this particular task.

**FIGURE 5:**
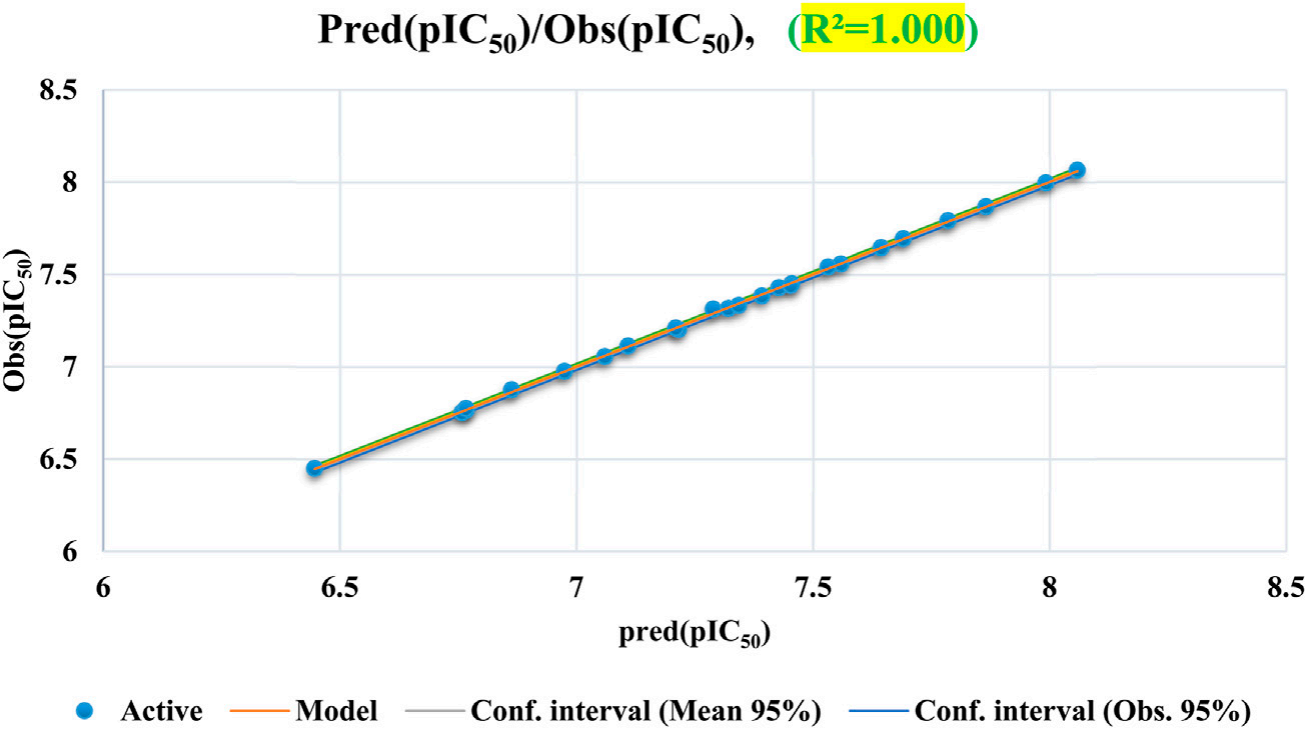
Correlation between the observed and the predicted activities calculated by ANN.

Figure 5 demonstrates a uniform distribution of predicted pIC_50_ values within the training set, indicating that the ANN model effectively correlates with experimental pIC_50_ data.

### Cross-validation (CV)

**FIGURE 6:**
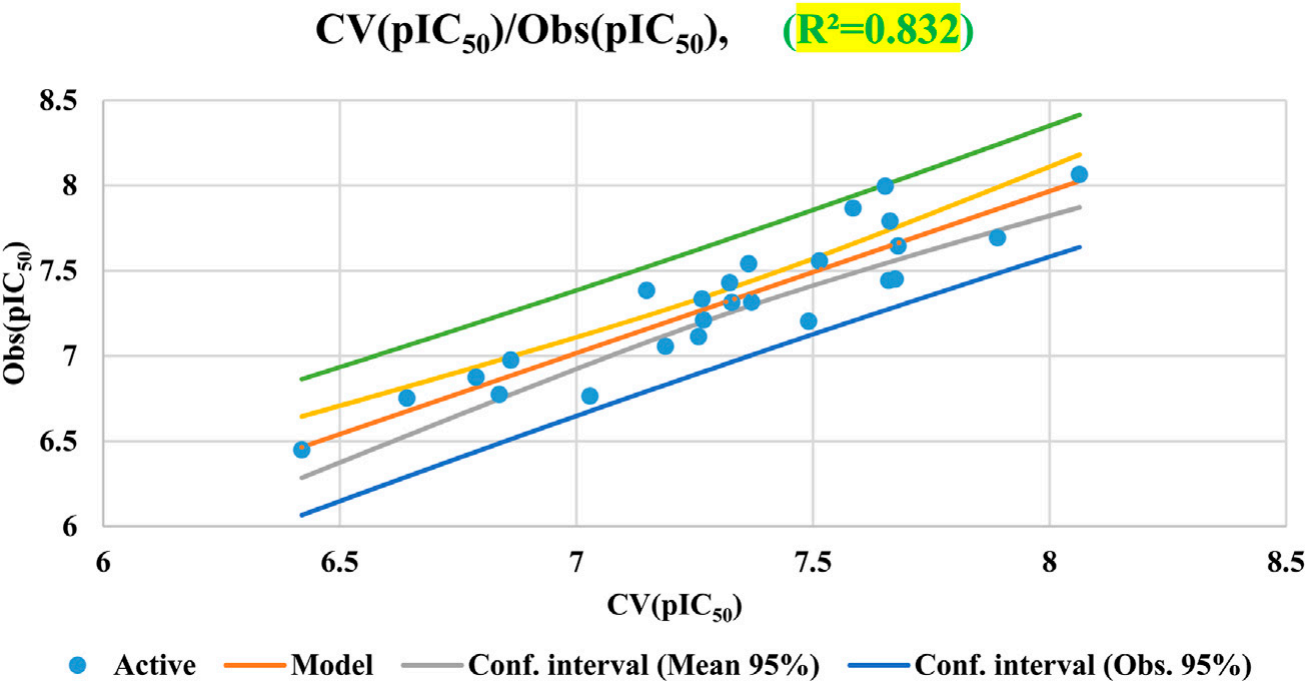
Correlation of observed and predicted activities calculated using LOO-CV.

Results of cross-validation based on the Leave-one-out (LOO) approach are shown in Figure 6. The cross-validation procedure does not significantly affect the developed QSAR model, as indicated by the derived parameters, *R* ^2^ = 0.83 and MSE = 0.027. These unambiguous findings show how stable and reliable the suggested QSAR model is. It is important to note that cross-validation alone may not be sufficient for fully evaluating the predictive capabilities of QSAR models.

### External validation

**FIGURE 7:**
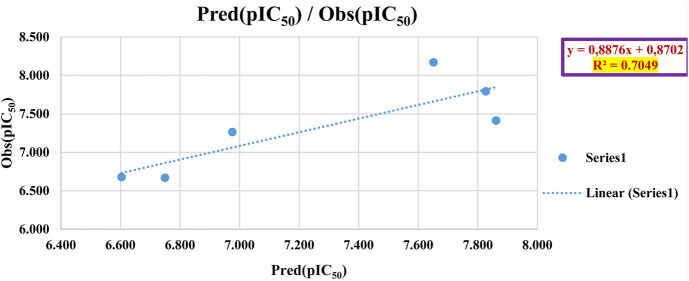
Correlation analysis of predicted vs. observed activity for the test set (MLR model).

To assess the predictive capability of the QSAR models, we performed external validation using the Golbraikh-Tropsha criteria (Table 4) (Golbraikh et al., 2003) his involved evaluating the model’s ability to accurately predict pIC_50_ values for molecules within the test set as resulted in Table 5. To assess the model’s predictive power, we calculated the correlation coefficient *R* ^2^. A higher *R* ^2^ value indicates a stronger correlation between the predicted and actual activities of the molecules, suggesting a more effective model, as shown in Figure 7.

**TABLE 4:** Criteria of Golbraikh and Tropsha’s for external validation.

Parameter	Threshold	Modelscore
$Q_{\text{training}}^{2}$	$Q_{\text{training}\;}^{2}$ > 0.5	0.618
$r^{2}$	$r^{2}$ > 0.6	0.7049
$r_{0}^{2}$		0.6922
$r_{0}^{\prime 2}$		0.661
|r_0_^2^- r’_0_^2^|	|r_0_^2^- r’_0_^2^| < 0.3	0.0382
K	0.85 < k < 1.15	1.00
$\frac{r^{2}-r_{0}^{2}}{r^{2}}$	$\frac{r^{2}-r_{0}^{2}}{r^{2}}<0.1$	0.018
$k^{\prime }$	0.85 < k’ <1.15	0.99
$\frac{r^{2}-r_{0}^{\prime 2}}{r^{2}}$	$\frac{r^{2}-r_{0}^{\prime 2}}{r^{2}}<0.1$	0.062

**TABLE 5:** Predicted activities of test set compounds using MLR.

Compounds	pIC_50_ obs	Pred (pIC_50_) MLR	Residual
3	6.750	6.668	0.082
4	6.604	6.677	−0.073
12	7.652	8.170	−0.518
21	6.976	7.263	−0.287
24	7.862	7.412	0.450
29	7.827	7.793	0.034

The QSAR model demonstrated high predictive accuracy, with an *R* ^2^ value of 0.7049 falling within the acceptable range. This indicates successful validation according to the Golbraikh and Tropsha criteria. Additionally, external validation confirmed the model’s ability to accurately predict pIC_50_ values for antiproliferative activity against MCF-7 cancer cells.

### Applicability domain (AD)

**FIGURE 8:**
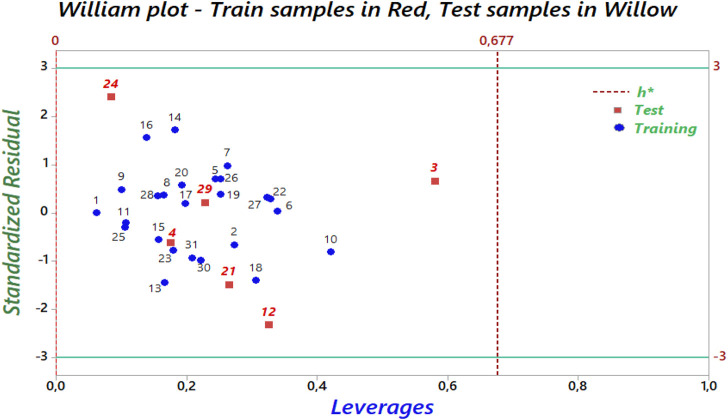
The Williams graph of the model presented by Equation 5.

The William plot for the AD of the model is shown in Figure 8.

The Williams plot (Figure 8) illustrates the application of leverage analysis to define the applicability domain (AD) of the QSAR model. According to the results of the Williams diagram, the leverage values are below the warning leverage (h*) for all compounds in the training and test sets. The warning leverage is calculated using the formula h* = 3 (p + 1)/n, where p represents the number of model parameters, and n denotes the number of compounds. In this case, h* is equal to 0.677. The lack of outliers in the test sets enabled the QSAR model to generate precise predictions. Consequently, all tested chemicals fall within the AD, validating the anticipated activity levels. These findings confirm the reliability and robustness of the developed QSAR model.

### Molecular docking simulations

**FIGURE 9:**
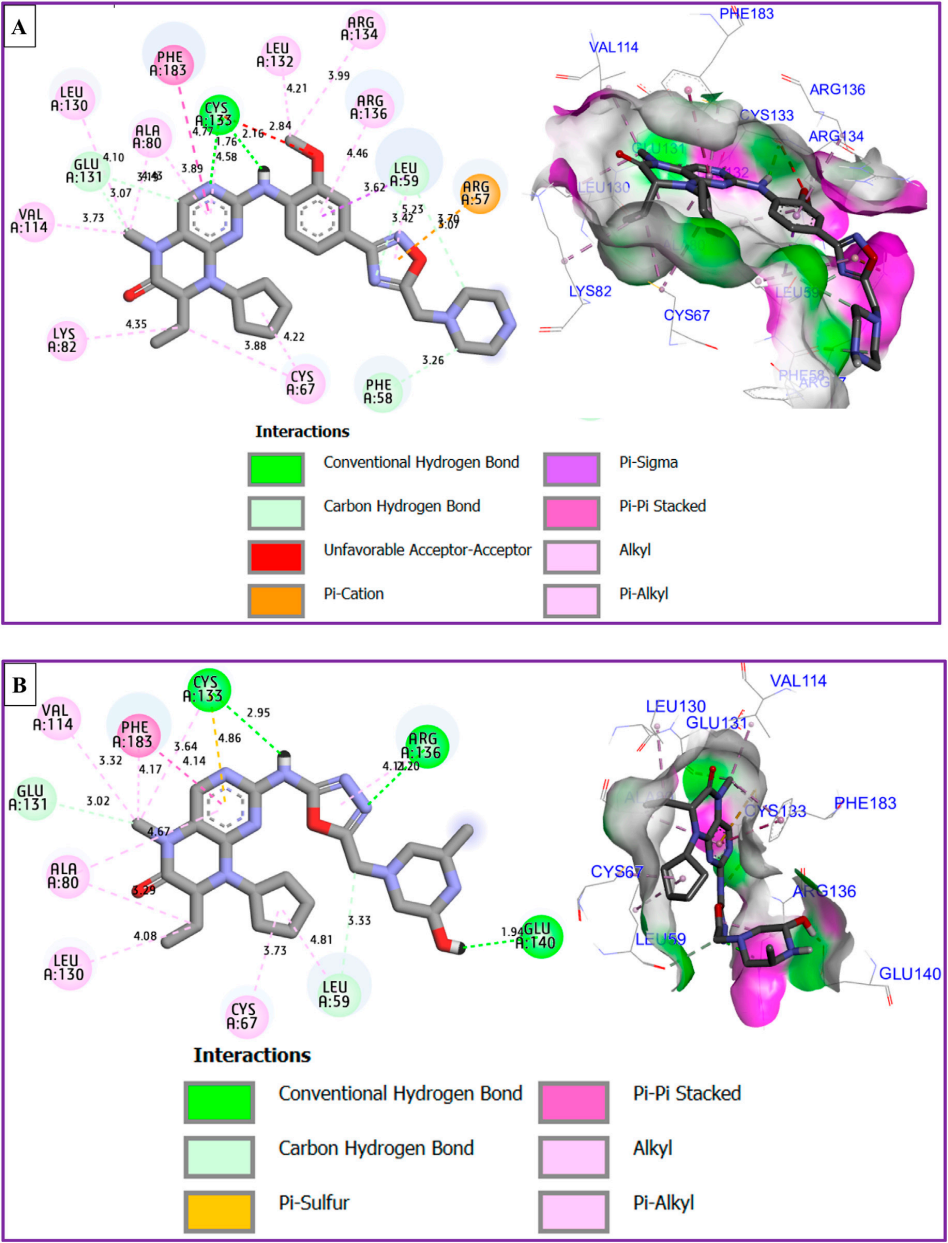
2D and 3D views of intermolecular contacts between the targeted protein of MCF-7 breast cancer cells (PDB ID of 2RKU) in complex with M28 **(A)** and M5 **(B)** molecules, respectively.

The results of molecular docking reveal a variety of intermolecular interactions that were produced between the most active compound labeled C28 and the targeted protein of MCF-7 breast cancer cells coded as 2RKU.pdb such as one Hydrogen bond detected with Cys133 amino acid residue, more than one Pi-Cation bond fixed with Arg57 amino acid residue, one Pi-Pi Stacked bond created towards Phe183 amino acid residue, in addition to several Alkyl and Pi-Alkyl bonds, as presented in Figure 9A.

The best candidate ligand known by M5, was equally docked to the structure of PLK1 in complex with BI2536 (PDB ID of 2RKU) sharing common intermolecular interactions like those detected towards Phe183, Cys133, Ala80, Leu130, Glu131, Cys67, and Arg136 amino acids residues as presented in Figure 9B. Moreover, a fairly low energy level in kcal/mol was observed for both conditioned molecules, justified by a binding energy of −8.25 kcal/mol and −6.38 kcal/mol for M28 and M5, respectively.

### Molecular dynamics simulations

**FIGURE 10:**
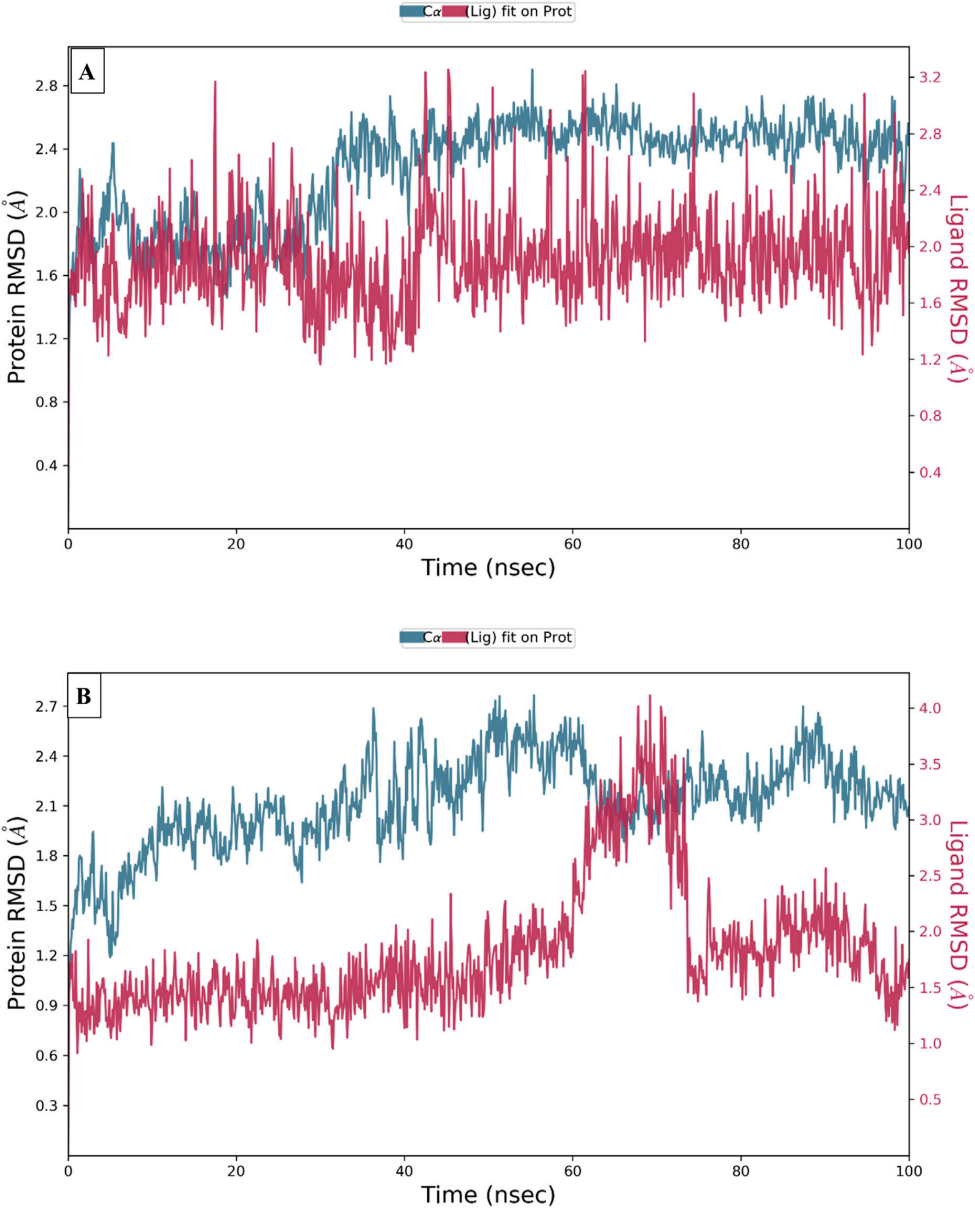
Conformational changes in RMSD values during 100 ns of MD time for the targeted protein of MCF-7 breast cancer cells (PDB ID of 2RKU) in complex with M28 **(A)** and M5 **(B)**, respectively.

**FIGURE 11:**
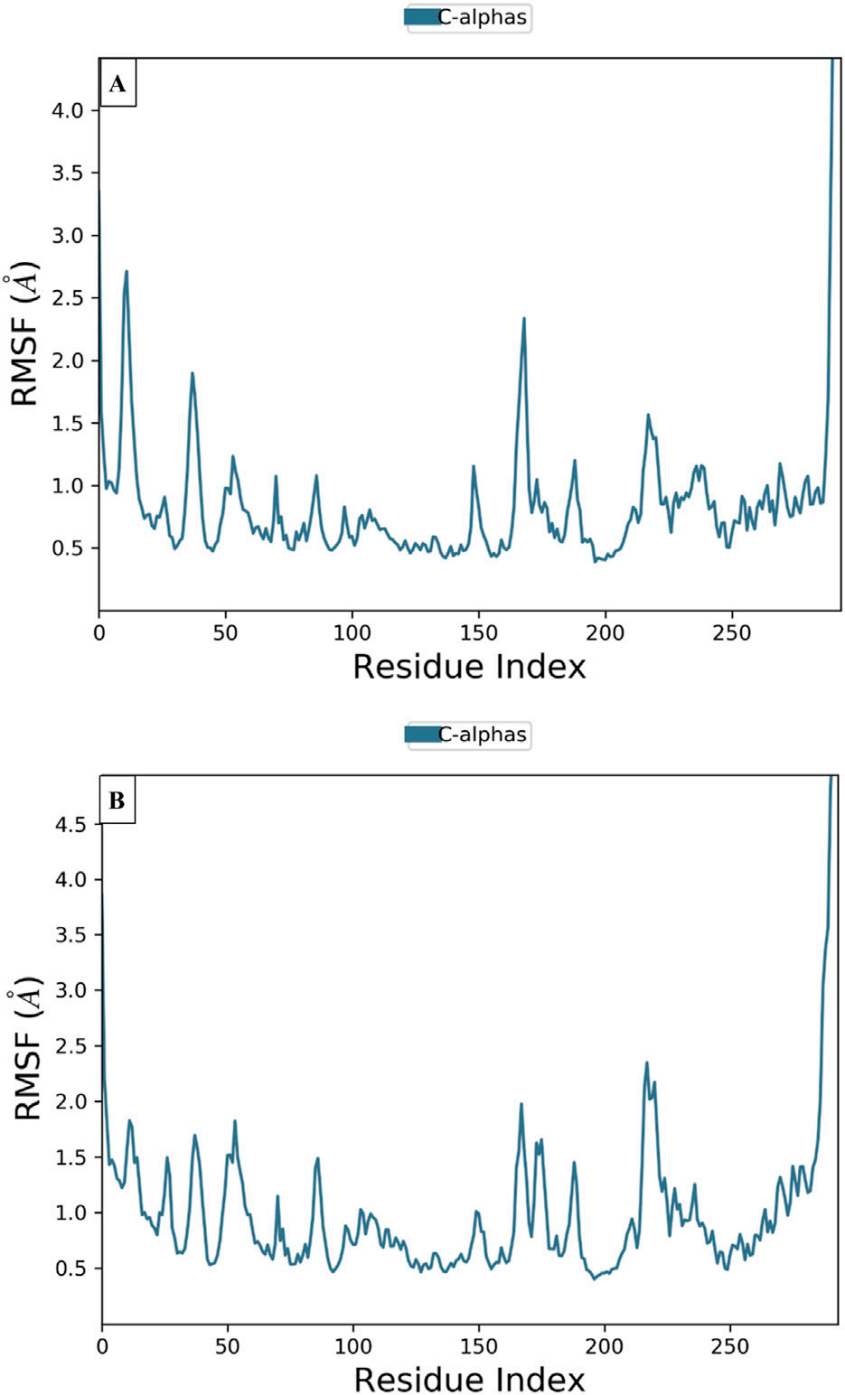
Conformational changes in RMSF values for the targeted protein of MCF-7 breast cancer cells (PDB ID of 2RKU) in complex with M28 **(A)** and M5 **(B)**, respectively.

**FIGURE 12:**
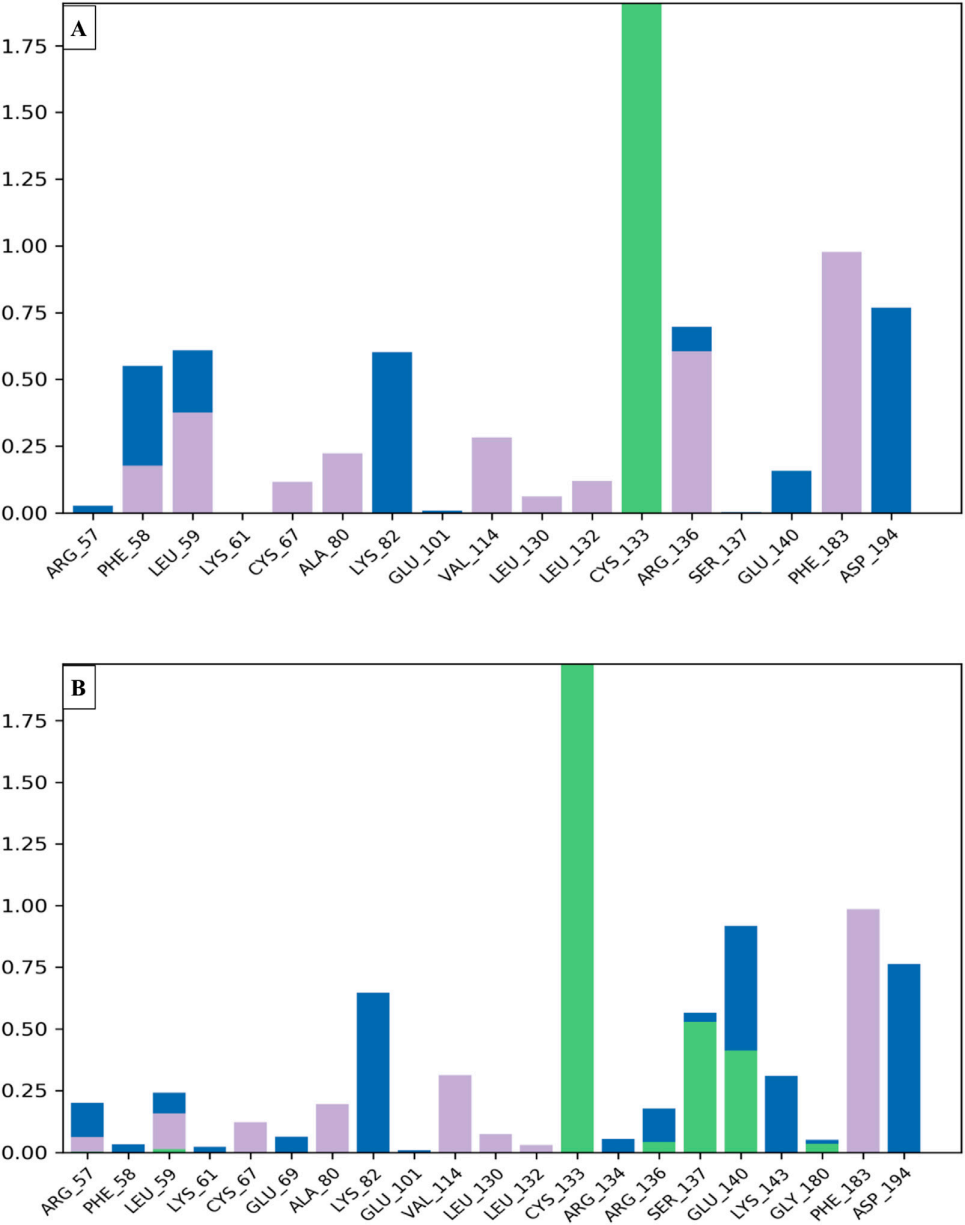
Interaction fractions by the MCF-7 breast cancer cell-targeted protein (PDB ID of 2RKU) interacts with compounds M28 **(A)** and M5 **(B)** via hydrophobic interactions represented by purple bars, water-bridge interactions represented by blue bars and hydrogen bonds represented by green bars.

To examine the thermodynamic stability of intermolecular interactions produced by molecular docking technique for M28 and M5 molecules in complex with the targeted receptor of MCF-7 breast cancer cells, the conformational changes in root mean square deviation (RMSD) and root mean square fluctuation (RMSF) were controlled throughout 100 nanoseconds of molecular dynamic’s simulation time, in which the obtained results indicate that M28 ligand synthesized by the lowest inhibitory activity was complexed to the protein target revealing a good level of molecular stability over 100 nanoseconds, which is justified by smaller RMSD and RMSF values that not exceed 3Å threshold (Er-rajy et al., 2022a; Er-rajy et al., 2023; Bouzammit et al., 2024). Almost the same level of thermodynamic stability was detected for the M5 ligand as the most active molecule among all five novel synthesized compounds, in which all corresponding RMSD and RMSF values oscillated in an equilibrium that did not exceed 3Å threshold as presented in Figures 10, 11, respectively. In other side, the interaction fractions diagram confirms that Phe183, Ala80, Leu130, Glu131, Cys67, and Arg136 amino acids residues have an important rule and especially Cys133 that was strongly implicated in the intermolecular contacts of the responsible receptor of MCF-7 breast cancer cells towards both M28 and M5 ligands, as displayed in Figure 12.

### Design of new compounds

The main aim of this work is to design new MCF-7 inhibitors based on dihydropteridone containing oxadiazoles, considering the insights gathered from the 2D-QSAR studies. In this regard, four dihydropteridone derivatives (M1, M2, M3, M4 and M5) were designed to improve the inhibitory activity of the MCF-7 inhibitor (Table 6). Using the same method employed for the previously studied molecules, we calculated the descriptors for these newly designed compounds. The RLM model was then used to predict their activity, as summarized in Table 7, the newly designed candidate compounds exhibit significantly higher inhibitory activity than the most active compounds in the studied series. These findings suggest that the designed compounds have the potential to serve as more effective MCF-7 inhibitors.

**TABLE 6:** Predicted pIC_50_ Values and Structural Features of Novel Compounds.

Compounds	Structure	pIC_50__pred
M1	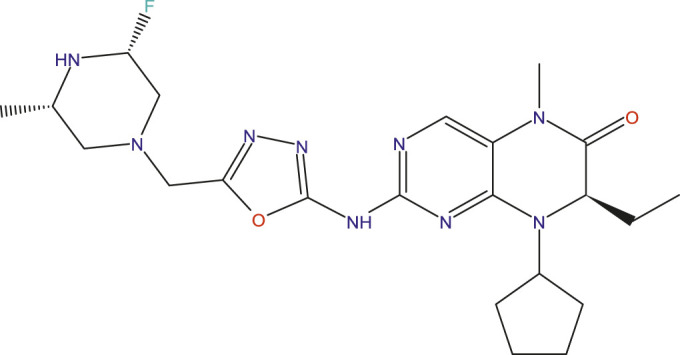	9.28
M2	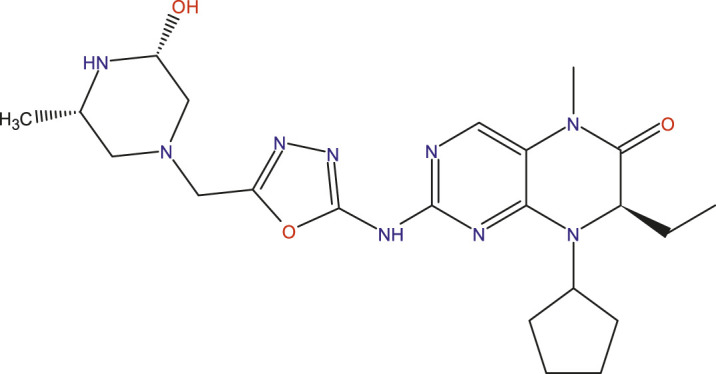	9.53
M3	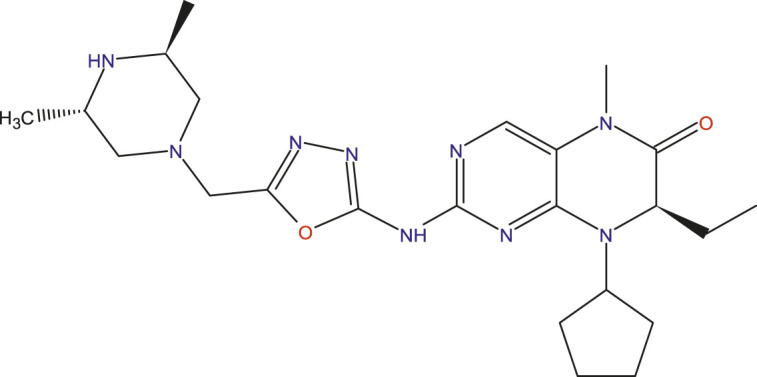	9.38
M4	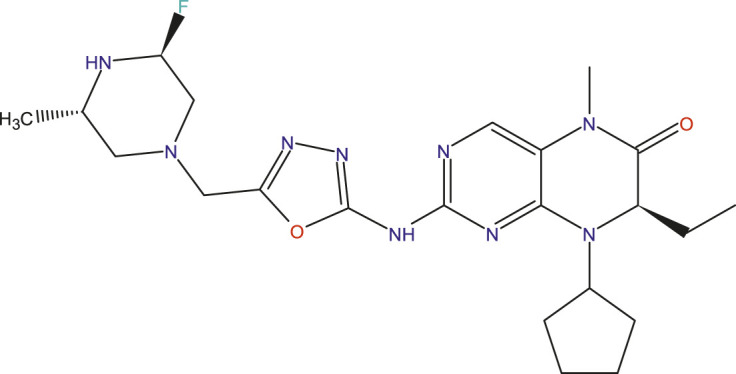	9.42
M5	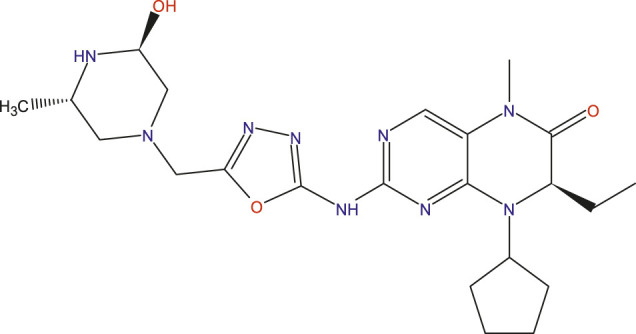	9.58

**TABLE 7:** The values of parameters calculated for the news molecules and this predict activity.

Comp	S	Tor	MP	NRB	repul	η	pIC50_pred
M1	2.3184	10.7868	1106.02	6	3659.74	0.08601	9.28
M2	2.281	12.669	1166.25	6	3579.79	0.086	9.53
M3	2.4091	10.8049	1116.7	6	3640.16	0.08575	9.38
M4	2.3513	10.94	1106.02	6	3690.36	0.08685	9.42
M5	2.3358	11.5044	1166.25	6	3647.79	0.08576	9.58

### Lipinski’s rule

All the proposed new compounds adhere to Lipinski’s rules (Table 8). The fact that the compounds adhere to Lipinski’s rule of five suggests satisfactory physicochemical characteristics, meaning that they have the potential for good oral bioavailability and may be candidates for marketing authorization. However, it is essential to stress that Lipinski’s rule of five is a recommendation, not a definitive rule. Further preclinical studies are necessary to thoroughly evaluate these compounds’ suitability as potential medications.

**TABLE 8:** Drug-like properties of novel compounds.

	TPSA	n-RotatableBonds	MW	LogP	n-HBA	n-HBD	Violations				Synthetic accessibility	Toxicity
							Lipinski	Veber	Egan	Muegge		AMES toxicity
Rule	<140 A^2^	<10	<500 Da	≤5	<10	<5	≤2	≤2	≤2	≤2	0 < *S.A*<10	Categorical (yes/no)
M1	115.55	6	473.55	3.98	9	2	Yes	Yes	Yes	Yes	5.12	No
M2	135.78	6	471.56	3.47	9	3	Yes	Yes	Yes	Yes	5.09	No
M3	115.55	6	469.58	3.98	8	2	Yes	Yes	Yes	Yes	5.15	No
M4	115.55	6	473.55	3.80	9	2	Yes	Yes	Yes	Yes	5.12	No
M5	135.78	6	471.56	3.83	9	2	Yes	Yes	Yes	Yes	5.09	No

### ADMET properties

To assess the potential suitability of the designed molecules as medications, we evaluated their pharmacokinetic properties, specifically ADMET (Absorption, Distribution, Metabolism, Excretion and Toxicity). The *in silico* ADMET properties were predicted using the pkCSM online tool (Pires et al., 2015), and the results are shown in the table below (Table 9).

**TABLE 9:** ADMET properties.

Models	Properties											
	Absorption		Distribution	Metabolism							Excretion	Toxicity
	Intestinal absorption (human)	P-Gp substrate	VDss (human)	CYP450							Total clearance	AMES toxicity
				Substrate		Inhibitor						
				2D6	3A4	1A2	2C19	2C9	2D6	3A4		
Unity	Numeric (%absorbed)	Categorical (yes/no)	Numeric (Log L kg^−1^)	Categorical (YES/NO)							Numeric (log mL min^−1^ kg^−1^)	Categorical (yes/no)
Predicted values												
M1	87.998	Yes	0.617	No	Yes	No	No	No	No	No	0.448	No
M2	79.198	Yes	0.593	No	Yes	No	No	No	No	No	0.456	No
M3	78.934	Yes	0.808	No	Yes	No	No	No	No	No	0.49	No
M4	87.998	Yes	0.617	No	Yes	No	No	No	No	No	0.448	No
M5	79.198	Yes	0.593	No	Yes	No	No	No	No	No	0.456	No

The data in Table 9 lead to a number of conclusions:

- A threshold below 30% indicates poor human intestinal absorption. All predicted molecules have an absorption value in excess of 79%, indicating that they are well absorbed by the human intestine.
- The volume of distribution (VDss) is a pharmacokinetic parameter that indicates the distribution of drugs between blood plasma and tissues. A low VDss indicates limited tissue distribution, while a high VDss suggests significant tissue distribution. Predicted VDss values for these compounds reveal their suitability for good tissue distribution (Ahmed, 2015). Additionally, the partition coefficient (LogP) values for all predicted compounds are below 4, suggesting favorable distribution between blood and tissues. However, these compounds are likely to have a poor degree of central nervous system (CNS) permeability, in accordance with their CNS Index LogP values above 3 (El fadili et al., 2023b).
- Cytochrome P450 (CYP) enzymes, present in all body tissues, play a crucial role in detoxification by oxidizing foreign compounds, These enzymes can both inhibit and activate various drugs, affecting their metabolism, Knowing whether a compound can inhibit CYP enzymes is essential for drug development. There are seventeen CYP families in humans, of which CYP1A2, CYP2A3, CYP2A4, CYP3A4, CYP2C9, CYP1A2, CYP2D6 and CYP2C19 are responsible for metabolizing over 90% of drugs undergoing 1st-pass metabolism (Šrejber et al., 2018). CYP3A4 and CYP2D6 are the two main isoforms involved in drug metabolism. According to the analysis of predicted compounds, these may be CYP3A4 substrates only (Chandrasekaran et al., 2018). This could affect the pharmacokinetics of these drugs, altering their elimination rate and onset of action.
- To optimize drug dosage and guarantee fixed concentrations, it is essential to take into account clearance rates, which depend on hepatic metabolism and renal excretion (Benkhaira et al., 2023). The lower the clearance index, the greater the drug’s half-life. Our analysis indicates that all predicted compounds have a total clearance index of less than 0.5, suggesting long retention in the body. This prolonged presence probably contributed to their efficacy in inhibiting MCF-7 cells, albeit at very low doses.
- Toxicity studies are an essential step in drug development. To assess the mutagenic potential of predicted compounds, *in silico* Ames tests were performed (Shinu et al., 2022). None of the compounds were predicted to be mutagenic. Additionally, the ADMET *in silico* evaluation confirmed that all the predicted molecules met the established pharmacokinetic criteria.

Given their potential to inhibit MCF-7 breast cancer cells, these compounds could serve as promising candidates for future cancer treatments. Furthermore, they can form the basis for the development of new molecules with enhanced biological properties and broad therapeutic applications.

## Conclusion

This study aimed to identify novel dihydropteridone derivatives with oxadiazole moieties as potential inhibitors of MCF-7 breast cancer cells. A 2D-QSAR analysis was employed to elucidate the structural determinants influencing biological activity. The MLR method yielded a robust QSAR model with exceptional predictive power. Key descriptors (S, Tor, MP, NRB, repul and η) were identified as critical for inhibitory activity. Based on the robust QSAR model, five promising compounds were rationally designed and subsequently subjected to *in silico* evaluation. Molecular docking studies demonstrated favorable binding interactions between these compounds and the target protein, suggesting potential for strong binding affinity. Furthermore, comprehensive pharmacokinetic and ADMET assessments indicated promising profiles for these compounds, including good absorption, reasonable distribution, and acceptable toxicity levels. These encouraging findings collectively suggest that the identified derivatives possess the potential to serve as promising lead candidates for the development of novel and efficacious anti-breast cancer agents. Future research efforts will focus on constructing 3D-QSAR models to gain a more nuanced understanding of the intricate structure-activity relationships within this chemical space. This deeper understanding will facilitate the design and optimization of even more potent and selective inhibitors, ultimately paving the way for the development of novel and improved therapeutic interventions for breast cancer.

## Data Availability

The original contributions presented in the study are included in the article/supplementary material, further inquiries can be directed to the corresponding authors.
